# Examining the Factors Associated with Paid Employment of Clients Enrolled in First Episode of Psychosis Programs

**DOI:** 10.1155/2012/739616

**Published:** 2012-06-21

**Authors:** Carolyn S. Dewa, Lucy Trojanowski, Chiachen Cheng, Desmond Loong

**Affiliations:** ^1^Centre for Research on Employment and Workplace Health, Centre for Addition and Mental Health, 455 Spadina Avenue, Suite 300, Toronto, ON, Canada M5S 2G8; ^2^Department of Psychiatry, University of Toronto, 250 College Street, Toronto, ON, Canada M5T 1R8; ^3^Clinic & Resource Centre, Canadian Mental Health Association, 272 Park Avenue, Thunder Bay, ON, Canada P7B 1C5

## Abstract

Schizophrenia is one of the most debilitating mental disorders. For a significant portion of individuals who suffer from this disorder, onset occurs in young adulthood, arresting important social and educational development that is necessary for future successful labor force participation. The purpose of this paper is to contribute to the literature about clients enrolled in first episode psychosis programs and psychosocial outcomes by examining the factors associated with paid employment among young adults who have experienced their first psychotic episodes. In this paper, we consider the association of socioeconomic factors to employment. Our results suggest that in addition to treatment, socioeconomic factors such as receipt of public disability benefits and educational attainment are associated with employment status. These results can help to inform future directions for the enhancement of psychosocial programs in FEP models to promote paid employment.

## 1. Introduction

Schizophrenia is one of the most debilitating mental disorders. For a significant portion of individuals who suffer from this disorder, onset occurs in young adulthood, arresting important social and educational development that is necessary for future successful labor force participation. As a result, schizophrenia is often associated with an eventual downward spiral ending in poverty and isolation[1]. To avert this dismal future, the focus on the first psychotic episode (FEP) is becoming a priority for mental health care globally (e.g., Johannessen et al. [2]).

There is also growing evidence surrounding the low employment rates of people with FEP. Employment rate estimates range from 13 to 55% [3–5]. Reviews of the literature [3, 6] note that there are a number of factors associated with employment along a number of dimensions [7–9]. Along with cognitive impairment and symptoms, educational attainment, family socioeconomic status, social benefit structures, and labour market conditions also potentially affect employment of young adults with first episode psychosis [3]. There appears to be a complex mix of both individual and environmental factors linked to successful labour market participation. Although it is a multifaceted challenge, there is increasing evidence for the effectiveness of interventions such as Individual Placement and Support (IPS) programs and the interventions of vocational specialists for this population [4, 6, 10–13]. As such, the International First Episode Vocational Recovery (iFEVR) Group [14] released a consensus statement advocating for the “right to education, training and employment” for young people enrolled in early intervention programs. 

The need for the iFEVR consensus statement is motivated by the mounting evidence that employment is often not a priority for providers who serve and support FEP clients. For example, Rinaldi and colleagues [6] note that few FEP studies have focused on employment and education outcomes. In fact, employment is often discouraged by well-intentioned medical professionals and family [6, 10, 15]. In addition, governments have been less than supportive of employment programs for people with mental illnesses [16]. 

The purpose of this paper is to contribute to the literature about clients enrolled in first episode psychosis programs and psychosocial outcomes by examining the factors associated with paid employment among young adults who have experienced their first psychotic episode. In this paper, we consider the association of socioeconomic factors with employment.

## 2. Background

### 2.1. Characteristics of FEP Programs

FEP programs are designed to facilitate recovery from psychotic illnesses that first arise during youth, most commonly schizophrenia and bipolar disorder, and have historically been most associated with very high levels of disability [17]. Programs are designed to provide services to both clients and their families. Services include comprehensive diagnostic assessment, treatment, psychosocial supports, as well as family education and support. There is emphasis on a multidisciplinary team approach that integrates medical treatment, counseling, case management, substance abuse treatment, cognitive behavioural therapy, in addition to psychoeducation for families. 

### 2.2. Characteristics of the Study FEP Programs

The six FEP programs included in this study were located in six regions throughout the province of Ontario, Canada. Each program accepted youth who were experiencing their first episode or early stages of psychosis. Each early intervention program was developed to exclusively provide outpatient services. 

Five of the six FEP programs reported that nearly three-quarters of their clientele were males. Three of the FEP programs engaged clients aged 14 to 35 years old, while the remaining three programs limited access to people who were at least 16 years old. Two FEP programs engaged transitional aged youth 16 to 23 years old. 

All but one of the six programs were located in established community mental health agencies. The exception was a community-based program that was part of an acute care hospital. The number of staff members in each of the six programs varied considerably, ranging from three part-time positions to 10 full-time equivalent positions. Each of the FEP programs was developed in accordance with the guidelines and standards set forth by the International Early Psychosis Association and other pioneers in the field [17–20].

### 2.3. FEP Programs and Vocational Outcomes

There is evidence that compared to FEP clients who receive usual care, those enrolled in early intervention programs have significantly better educational and vocational outcomes [21]. The relatively better vocational outcomes associated with FEP programs may be associated with the shorter duration of untreated psychosis for these clients. Norman et al. [22] found that shorter duration of untreated psychosis and greater social support were significantly associated with either more full-time competitive work or full-time enrollment in school at three-year followup. 

In their review, Rinaldi and colleagues [6] note that when compared to those enrolled in community mental health teams, clients enrolled in FEP program seem to experience a relatively smaller decline in employment and education. They attribute this to a protective role played by FEP programs. Major et al. [5] observed a decrease in unemployment among FEP clients after 12 months even in the absence of a vocational focus.

However, there is less evidence regarding the long-run outcomes for FEP programs versus usual care. After 18 months, the LEO (Lambeth Early Onset team) study did not find [21] a significant difference between clients in specialize programs versus those who were not. After 5 years, the OPUS trial did not find significant differences in employment between clients who were in FEP versus usual care [23]. 

### 2.4. Work History and Employment

In the literature focusing on vocational outcomes in the adult population with severe and persistent mental disorders, it has been observed that work history is one of the most consistent predictors of employment [24]. However, because of their stage in life, young adults experiencing their first psychotic episode have not had the opportunity to accumulate labor force experience. This can place FEP clients at a disadvantage in finding paid employment.

### 2.5. Educational Attainment and Employment

The literature also indicates that educational attainment is significantly related to employment status. People who have no high school diploma are more likely to be unemployed [25, 26]. If young people have their education interrupted by their illness, they can be faced with another disadvantage in finding employment.

### 2.6. Disability Benefits and Employment

There is also an association between receipt of disability benefits and unemployment [10, 27]. The fear of losing disability benefits has been identified as one of the barriers to obtaining and maintaining employment for clients with severe mental illness [3, 24, 28]. As a result, those who receive benefits often have poorer employment experiences. 

Thus, there is accumulating evidence suggesting that in addition to treatment in FEP programs, socioeconomic factors such as educational attainment and income sources also play a role in employment outcomes. This type of evidence can help to inform future directions for the enhancement of psychosocial programs in FEP models.

## 3. Methods

### 3.1. Data Collection

The study protocol was approved by the Centre for Addiction and Mental Health's Research Ethics Board. A cross-sectional data collection approach was used at three points in time during October 2005, 2006, and 2007 in six FEP programs located throughout Ontario, Canada's largest and most populous province. 

Interview participation criteria included (1) willingness to be contacted by a study interviewer, (2) ability to understand and give informed consent to be interviewed, and (3) enrollment in one of the six participating FEP programs. Data were collected using face-to-face structured interviews administered by trained interviewers. Potential study participants were referred by program case managers. 

In 2005, the participating FEP programs referred 45 clients (28%) of the total 161 enrolled, to be contacted by the study; of these, 33 (73%) were successfully interviewed. They represented 20% of the total clients enrolled. In 2006, 106 (32%) of 302 early intervention clients were eligible to be contacted by the study. There were 75 (71%) who were successfully interviewed. They represented 25% of the total clients. In 2007, 162 (44%) of 370 early intervention clients were eligible to be contacted for interviews. Of these, 107 (66%) were successfully interviewed. These clients constituted 29% of the total early intervention clients enrolled in the six programs.

### 3.2. Dependent Variable

An indicator variable was created to capture whether or not study participants had paid employment during the previous 12 months. 

### 3.3. Study Participant Characteristics

Indicator variables were created to capture whether the respondent was living with family (i.e., parent(s), sibling(s), and spouse), education (completed high school: yes/no), gender (male: yes/no), age at time of interview, receipt of provincial disability benefits as the primary income source (yes/no) and enrolled in school during the previous 12 months (yes/no). An indicator variable was also created to capture whether participants were enrolled in their FEP program for more than a year. 

Three region indicator variables were created to capture the population density of the region in which the participant lived. Region 1 indicated that the participant resided in a region in which the population density was less than 100 people/km^2^. Region 2 indicated that the region of residence had a population density between 200 and 450 people/km^2^. Finally, Region 3 indicated that the region of residence had 3,929 people/km^2^. 

### 3.4. Analyses

Descriptive statistics were used to test for significant differences in the characteristics of people who had paid employment during the previous 12 months. Chi-square and Fisher exact tests were used to test the differences between the categorical variables. 

Logistic regression analysis was used to identify the factors associated with having had paid employment. The most parsimonious model was used based on the factors described in the literature that are associated with employment for people with serious mental illnesses. 

## 4. Results

About 61% of the sample had paid employment during the previous 12 months. In Table 1, compared to those who did not have employment, among those who had employment, there was a significantly greater proportion of people with at least a high school diploma (*χ* ^2^(2) = 6.21, *P* = 0.013) and a significantly lower proportion who identified provincial disability benefits as their primary income source (*χ* ^2^(2) = 23.31, *P* < 0.0001).

Table 2 contains the results of the logistic regression analysis. The regression model produced 83.2% concordant and 16.6% discordant predicted probabilities and observed responses. The Hosmer and Lemeshow Goodness-of-Fit Test was *χ* ^2^(8) = 10.99, *P* = 0.202. These results indicate that the null hypothesis cannot be rejected; the model fits the data and the model has adequate goodness-of-fit.

The results suggest that the odds of having been employed in the past 12 months are significantly lower for people who have not completed high school (OR = 0.34, 95% CI = 0.12, 0.97) and for clients who identified provincial disability benefits as their primary income source (OR = 0.069, 95% CI = 0.022, 0.21). There is also a significant positive association between employment and being enrolled in the FEP program for more than a year (OR = 3.014, 95% CI = 1.027, 8.85).

## 5. Discussion

In our sample, 61% of respondents had paid employment during the previous 12 months. This proportion is greater than that reported in the literature. For example, Singh et al. [29] observed that among the cohort of FEP clients, the past year employment rate (either full or part time) was 31.3%. Similarly, Norman et al. [22] reported employment rates of 44%, while Turner et al. [30] reported an employment rate of 46%.

We also observed a significant difference between the educational attainment of those who were employed and those who were not. Educational attainment is a significant concern among FEP clients [10, 11]. Norman et al. [22] observed that 46% of clients in their sample had attained less than a high school education at time of program entry. Assessing the three-year outcomes of FEP clients, Singh et al. [29] reported that 53.6% participants had not attained their secondary education certificates.

Our regression results underscore one of the major problems associated with the lack of educational attainment; educational attainment is associated with the ability to attain employment. Our results indicate that those who did not complete high school were less likely to be employed. This association corroborates what has been reported in literature [5, 31]. These results echo the need highlighted by Cook [32] in her review of the literature that a significant proportion of people with serious and persistent mental disorders have their education interrupted by the onset of mental disorders. That is, people with severe mental illness are likely to have lower educational attainment than their counterparts who have not experienced a mental disorder. In turn, this places them at a disadvantage to successfully compete in the labor market.

One of the arguments for early labour market participation is because of its effect on future employment. In their longitudinal study of people with severe mental illness, Bush and colleagues [33] found that those who were employed at the beginning of a 10-year period were more likely to remain steadily employed throughout. In contrast, those who were not employed at the beginning were more likely to remain unemployed throughout the period. It should be noted that Bush et al. [33] looked at a group of people with severe mental illness. It will be important for future studies to examine effects of early employment in the FEP population.

Our regression results also indicate that enrollment in a FEP program for more than a year is significantly associated with employment. That is, clients who have been enrolled in a FEP program for more than a year are three times more likely to have been employed during the year than clients who were enrolled for less than a year. This is a trend that has been reported in the literature. After a three-year followup of FEP clients, Singh et al. [29] saw past 12-month employment rates increase from 25.3% at intake to 31.3% at followup. This could be reflective of the protective effect provided by FEP programs [6].

None of the FEP programs that participated in this study had vocational specialists. This suggests that vocational outcomes were not necessarily one of the main foci of the programs. Yet, there was improvement in vocational outcomes. At the same time, compared to FEP programs without a vocational focus, those with IPS programs and vocational specialists have reported relatively better vocational outcomes [4, 6, 10–13]. Thus, although FEP programs appear to provide a protective effect, there is evidence that with a vocational focus, they can be more than protective. 

Finally, these results suggest that enrolment in a public disability benefit program also decreases the likelihood of being employed. Our results may reflect the fact that the people who receive disability benefits are too ill to be employed. Alternatively, the results could also be related to incentives associated with receipt of disability benefits. In her review of the literature, Cook [32] points out that few people leave public disability benefits due to employment. Our finding corroborates findings that disability benefits are inversely associated with employment rates. That is, as disability benefit levels decrease, employment rates increase, and the use of unemployment benefits decreases [34]. This suggests that publicly funded benefits may inadvertently create disincentives to work. For example, people might perceive that they will be penalized for working. In Ontario, the province in which this study was conducted, when people are employed and also receive benefits from the provincial disability support program, the program calculates half of the client's net monthly earnings, deducts part or all of his/her monthly child care and disability-related work costs, and subtracts this amount from the client's total income support [35]. Although in theory the earned income should substitute for income support, people who receive disability benefits and who work will see their disability income support decreased but not necessarily associate the decrease with their pay check.

At the same time, the provincial disability benefit program also seeks to create an incentive for paid employment by offering clients an additional monthly $100 work-related benefit [35] if they are employed in a paid position. Yet, the incentive to work may not be sufficient if people are unsure about their long-term ability to maintain employment. There may still be a fear that if they lose their disability benefits, they will be without a safety net if they lose their jobs [36–38]. It will be important for future research to explore the mechanisms of designing safety nets that ensure that when people are unable to work, they will have income while encouraging employment when it is possible. It will also be a challenge for FEP programs to design programs that help clients to receive training for jobs in business sectors in which they can earn a living wage [32] and that can accommodate episodes of illness so clients can accumulate successful employment histories.

**Limitations**

The results of this study should be considered in the light of its limitations. One of its major limitations relates to its generalizability. The clients who participated in these interviews may not necessarily be representative of all clients in FEP programs. Given that only clients who were able to provide informed consent were asked to participate, those who were the most sick would have been omitted. Thus, our results are a conservative estimate of the proportion who were unemployed if the most severely ill were more likely to be unemployed. Likewise, the regression results reflect associations for less severely ill people and may differ for the more severely ill; however, the latter group may also be less likely to be seeking employment.

In addition, the sample only included people who were enrolled in early intervention for psychosis programs. Results may be different for people experiencing their first psychotic episode who do not receive services from a program specializing in first psychotic episode cases.

## 6. Conclusions

There is little in the literature focusing on employment in the FEP population examining the contribution of socioeconomic factors to employment status. The results of our analyses indicate that receipt of public disability benefits and high school education are important factors related to employment. They also suggest that if paid employment is to be used as one measure of psychosocial outcomes, it is an outcome that may require cross-sector collaboration among health, education, and social services. 

## Figures and Tables

**Table 1:** Characteristics of FEP clients who did and did not have paid employment during previous 12 months.

Characteristics	Had paid employment during the previous 12 months		Did not have paid employment during the previous 12 months		Statistical tests for differences between characteristics of employed and unemployed
	% or mean	*n* or sd	% or mean	*n* or sd
Sex					
Male	70.2%	59	72.2%	39	*χ* ^2^(1) = 0.063, *P* = 0.80
Female	29.8	25	27.8	15	
Age (in years)	23.3	sd = 5.13	21.93	sd = 3.98	*t*-test (136) = −1.62, *P* = 0.11
No high school diploma	24.1%	20	44.4%	24	*χ* ^2^(1) = 6.21, *P* = 0.013
Population density of region of residence					
≤100 people/km^2^	52.4%	44	48.2%	26	*χ* ^2^(1) = 0.24, *P* = 0.63
200–450 people/km^2^	38.1	32	24.1	13	*χ* ^2^(1) = 2.94, *P* = 0.086
≥3,929 people/km^2^	9.5	8	27.8	15	*χ* ^2^(1) = 7.89, *P* = 0.0050
Living with family	66.7%	56	66.7%	36	*χ* ^2^(1) = 0.0, *P* = 1.0
Enrolled in school during the past 12 months	52.4%	44	40.7%	22	*χ* ^2^(1) = 1.78, *P* = 0.18
Provincial disability-benefits main source of income	17.7%	14	59.2%	29	*χ* ^2^(1) = 23.31, *P* < 0.001
Enrolled in FEP program >1 year	37.8%	31	28.3%	15	*χ* ^2^(1) = 1.29, *P* = 0.26

**Table 2:** Logistic regression results: outcome = paid employment during past 12 months.

Variables	Odds ratio	*P* value
Male (reference group: females)	0.78	0.64
Age (in years)	1.12	0.10
No high school diploma (reference group: has high school diploma)	0.34	0.045
Population density of region of residence		
(Reference group: <100 people/km^2^)		
200–450/km^2^	2.73	0.062
≥3,929/km^2^	1.21	0.78
Living with family (reference group: not living with family)	0.46	0.15
Enrolled in school during the past 12 months	1.29	0.62
(reference group: not enrolled in school in past 12 months)		
Provincial disability-benefits main source of income	0.069	<0.0001
(reference group: provincial disability benefits not main source of income)		
Enrolled in FEP program >1 year	3.014	0.045
(Reference group: enrolled in FEP program ≤1 year)		

*χ* ^2^(9) = 44.32, *P* < 0.0001		
*R* ^2^ = 0.30		
*n* = 124		
